# Chest computed tomography findings typical of COVID-19 pneumonia in Germany as early as 30 December 2019: a case report

**DOI:** 10.1186/s13256-023-03809-0

**Published:** 2023-03-24

**Authors:** Antonia Petersen, Sebastian Nagel, Bernd Hamm, Matthias Taupitz

**Affiliations:** Department of Radiology, Charité-Universitätsmedizin Berlin, Corporate Member of Freie Universität Berlin and Humboldt-Universität zu Berlin, Hindenburgdamm 30, 12203 Berlin, Germany

**Keywords:** COVID-19, Computed tomography, Europe, Germany, 2019, Case report

## Abstract

**Background:**

The first cases of coronavirus disease 2019 were officially confirmed in Germany and its European neighbors in late January 2020. In France and Italy, there is evidence that coronavirus disease 2019 was spreading as early as December 2019.

**Case presentation:**

We report on a 71-year-old male patient from Germany who was admitted to our hospital on 30 December 2019 with pneumonia of unclear etiology and chest computed tomography findings typical of COVID-19 pneumonia.

**Conclusion:**

This case may indicate that coronavirus disease 2019 was already spreading in Germany as early as December 2019.

**Supplementary Information:**

The online version contains supplementary material available at 10.1186/s13256-023-03809-0.

## Background

On 31 December 2019, the Wuhan Municipal Health Commission in Wuhan City, Hubei Province, China reported a cluster of pneumonia cases of unclear etiology [1]. On 9 January 2020, the identification of a novel coronavirus, later named severe acute respiratory syndrome coronavirus 2 (SARS-CoV-2), as the causative agent was announced by Chinese scientists [2]. On 30 January 2020, the World Health Organization (WHO) declared that the outbreak constitutes a Public Health Emergency of International Concern [3]. The first cases of coronavirus disease 2019 (COVID-19) in Europe were confirmed in France on 24 January 2020 in persons with a recent stay in Wuhan [4]. In Germany, the first case of COVID-19 was officially confirmed on 27 January 2020. This person had attended meetings in Germany with a Chinese business partner who tested positive for SARS-CoV-2 after she developed symptoms on her flight back to China [5].

We report on a 71-year-old male patient from Germany with respiratory symptoms who was in our care from 30 December 2019. Chest computed tomography findings suggested an atypical pneumonia. Retrospectively, the infiltrates show the characteristic appearance and distribution pattern of COVID-19 pneumonia.

The case was discovered when we were looking for evidence that SARS-CoV-2 had spread in Germany earlier than officially confirmed. For this purpose, we looked for findings typical of COVID-19 pneumonia in the chest computed tomography scans from December 2019.

## Case presentation

Admission to our hospital occurred on 30 December 2019 after the patient’s general condition had continuously deteriorated over 7 days following a fall. On arrival, the patient presented in a poor general condition. Auscultation revealed a reduced vesicular breath sound, bilateral rales, and an expiratory wheeze. Pulse oximetry showed an oxygen saturation of 90%. Heart rate was 92 beats per minute and blood pressure was 118/91 mmHg. The patient’s body temperature was increased to 37.8 °C. Blood analysis showed an elevation in white blood cell count (11,230/mm^3^) and c-reactive protein (39 mg/l). Creatinine was 1.66 mg/dl (146.1 µmol/l), estimated glomerular filtration rate (eGFR) according to the Chronic Kidney Disease Epidemiology Collaboration was 41 ml per minute per 1.73 m^2^ of body surface area. Known preexisting conditions included diabetes mellitus type II, hypertension, and hyperlipidemia. The patient was overweight. He had a history of smoking and suffered a stroke in the past. The patient had not been vaccinated against influenza. In the weeks before his hospitalization, he had been in regular contact with different people. He had not taken a trip abroad recently.

**Fig. 1:**
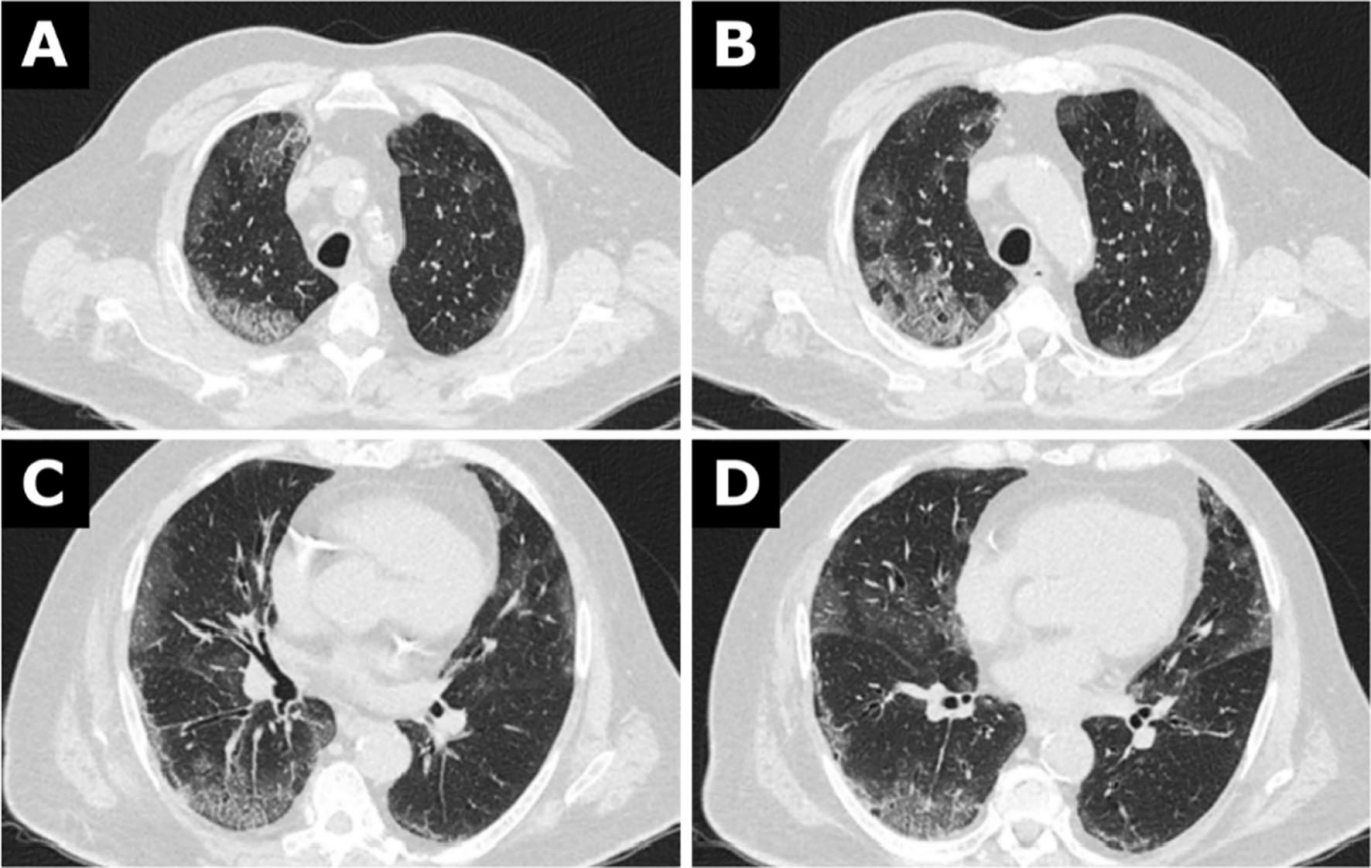
**A**–**D** Chest computed tomography from 30 December 2019

A contrast-enhanced computed tomographic scan of the head, neck, thorax, and abdomen was performed on the day of admission to exclude traumatic injury and to search for infectious foci. No traumatic injuries were found. The chest computed tomography (Fig. 1A–D) showed bilateral areas of ground-glass opacity with peripheral distribution. Both lower lobes were involved, and a posterior predilection could be seen. In parts, interlobular lines were visible resulting in a “crazy paving” pattern. Segmental and subsegmental vessels appeared dilated. There was no pleural effusion. The mediastinal lymph nodes were only slightly enlarged (Additional file 1 and 2). The computed tomography did not show evidence of other foci of infection. We diagnosed atypical pneumonia, including viruses as a possible causative pathogen.

Infection with *Legionella pneumophilia* and *Streptococcus pneumoniae* was ruled out, but no further pathogen diagnostics took place. Blood and urine cultures remained without pathogen detection. Initially, the patient received antibiotic treatment with ampicillin/sulbactam and clarithromycin. His condition was stable when 2–4 l of oxygen per minute were administered via nasal cannula.

**Fig. 2:**
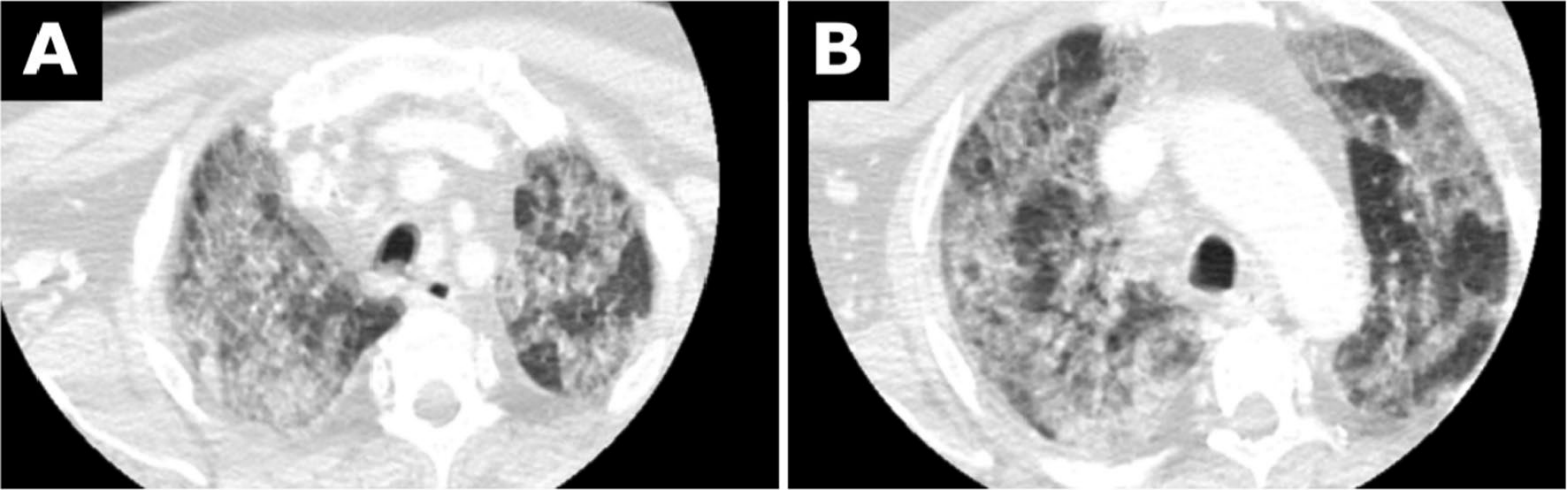
**A**, **B** Chest computed tomography images as part of head and neck computed tomography from 3 January 2020

Four days after admission, the patient showed new neurological symptoms. In the subsequent computed tomography scan, an occlusion of the right internal carotid artery was diagnosed. Progressive pneumonic infiltrates were seen in the included lung portions (Fig. 2A, B). During the attempt to recanalize the occluded right internal carotid artery, the patient required intubation and remained intubated for 5 days.

The inflammatory parameters reached a maximum 6 days after hospitalization (C-reactive protein 177 mg/l, white blood cell count 16,300/mm^3^). Under escalated antibiotic therapy, they decreased again. Later in the course, the patient also suffered from catheter-associated sepsis. Renal function recovered. Creatinine dropped to 0.75 mg/dl (66 µmol/l, eGFR > 90 ml per minute per 1.73 m^2^ of body surface area).

No further computed tomography was obtained. Chest radiographs showed decreasing pneumonic infiltrates. The patient was discharged home on 28 January 2020 with a severe persistent neurological deficit. He died in April 2020.

During his stay in hospital, the patient received regular visits from his family. One member of the family fell ill in early February 2020 and suffered from fever up to 41 °C for several days. A pathogen diagnosis did not take place.

## Discussion

If an infection is suspected, COVID-19 can be diagnosed in several ways [6], with reverse transcription polymerase chain reaction (RT-PCR) as recommended by the WHO as the preferred method [7]. Unfortunately, blood samples are no longer available from our patient for subsequent analysis.

Meta-analyses have shown that chest computed tomography has a sensitivity of over 90% in the case of patients experiencing symptoms of COVID-19 [8, 9], which was the case with our patient. His chest computed tomography findings match the typical appearance of COVID-19: peripheral, bilateral, ground-glass opacities with or without consolidation or visible intralobular lines (“crazy-paving”) [10]. Beyond that, vascular enlargement, bilateral abnormalities, lower lobe involvement, and posterior predilection are among the computed tomography abnormalities with a high incidence (> 70%) in RT-PCR test-proven COVID-19 cases [11]. There was no pleural effusion and no lymphadenopathy, which is also characteristic of COVID-19 [12].

Considering the chest computed tomography findings, it is likely that our patient is one of the earliest cases of COVID-19 in Germany. The clinical course is consistent with this assumption. Advanced age and male gender, as well as comorbidities, are more often associated with a more severe course [13]. High creatinine on admission is associated with a higher risk of in-hospital death [14]. Acute ischemic stroke severity in patients with COVID-19 is typically moderate at least (NIHSS score 19 ± 8), with a high prevalence (40.9%) of large vessel occlusion [15]. The high inflammatory parameters that the patient presented 6 days after hospitalization could have been caused by a bacterial superinfection.

This case suggests that COVID-19 was already spreading in Germany in December 2019. There is other evidence that SARS-CoV-2 was already spreading in Europe in December 2019. SARS-CoV-2-RNA was detected in wastewater samples in northern Italy as early as 18 December 2019 [16]. Wastewater-based epidemiology has proven to be a helpful tool for COVID-19 surveillance. In many places worldwide, the level of SARS-CoV-2-RNA in wastewater samples correlated with local COVID-19 incidence, preceding the increase of new clinical cases in the population by 1–3 weeks [17]. In France, RT-PCR was performed retrospectively on stored respiratory samples from December 2019. In one case dated 27 December 2019, the diagnosis of COVID-19 was confirmed [18].

As a limitation, it must be noted that a COVID-19 diagnosis could not be confirmed by RT-PCR for our patient. Differentially, an atypical pneumonia caused by another pathogen or other pathological conditions with similar chest computed tomography findings must be considered.

## Conclusion

The WHO has set itself the goal of investigating the origins of COVID-19 further. Retrospective examination of chest computed tomography for evidence of COVID-19 is listed as an appropriate means in this regard [19]. We present a patient who was admitted to our hospital on 30 December 2019 with pneumonia of unclear etiology, with chest computed tomography findings typical of COVID-19 pneumonia. This may indicate that COVID-19 was already spreading in Germany as early as December 2019.

## Supplementary Information

**Additional file 1.** Chest computed tomography from 30 December 2019.

**Additional file 2.** Chest computed tomography from 30 December 2019, lungs window.

## Data Availability

Not applicable.
